# 15-Deoxy-△^12,14^-Prostaglandin J_2_ Promotes Resolution of Experimentally Induced Colitis

**DOI:** 10.3389/fimmu.2021.615803

**Published:** 2021-02-02

**Authors:** Wonki Kim, Jeong-Hoon Jang, Xiancai Zhong, Hyungseok Seo, Young-Joon Surh

**Affiliations:** ^1^ Tumor Microenvironment Global Core Research Center and Research Institute of Pharmaceutical Sciences, College of Pharmacy, Seoul National University, Seoul, South Korea; ^2^ Department of Molecular Medicine and Biopharmaceutical Sciences, Graduate School of Convergence Science and Technology, Seoul National University, Seoul, South Korea; ^3^ Cancer Research Institute, Seoul National University, Seoul, South Korea

**Keywords:** cyclopentenone prostaglandin, resolution of intestinal inflammation, macrophage polarization, DSS-induced colitis, STAT3, 15-deoxy-△^12,14^-prostaglandin J_2_

## Abstract

Uncontrolled macrophage functions cause failure to resolve gut inflammation and has been implicated in the pathogenesis of inflammatory bowel disease (IBD). 15-Deoxy-Δ^12,14^-prostaglandin J_2_ (15d-PGJ_2_), one of endogenous lipid mediators formed from arachidonic acid during the inflammatory process, has been reported to terminate inflammation. However, the pro-resolving effect of 15d-PGJ_2_ on intestinal inflammation and underlying molecular mechanisms remain largely unknown. In the present study, we examined the effects of 15d-PGJ_2_ on the resolution of dextran sulfate sodium (DSS)-induced murine colitis that mimics human IBD. Pharmacologic inhibition of prostaglandin D synthase (PGDS) responsible for the synthesis of 15d-PGJ_2_ hampered resolution of inflammation in the colonic mucosa of mice treated with DSS. Notably, intraperitoneal injection of 15d-PGJ_2_ accelerated the resolution of experimentally induced colitis. 15d-PGJ_2_ treatment reduced the number of neutrophils and M1 macrophages, while it increased the proportion of M2 macrophages. Moreover, 15d-PGJ_2_ treated mice exhibited the significantly reduced proportion of macrophages expressing the pro-inflammatory cytokine, IL-6 with concomitant suppression of STAT3 phosphorylation in the colonic mucosa of mice administered 2.5% DSS in drinking water. Taken together, these findings clearly indicate that 15d-PGJ_2_, endogenously generated from arachidonic acid by cyclooxygenase-2 and PGDS activities in inflamed tissue, promotes resolution of intestinal colitis.

## Introduction

Inflammatory bowel disease (IBD), including ulcerative colitis and Crohn’s disease, is a chronic disorder characterized by inflammation of the gastrointestinal tract typically with a relapsing and remitting clinical course (1, 2). In IBD, the immune response is initiated by the interaction among the components of the innate immune system, including macrophages and dendritic cells (3). In particular, mucosal macrophages represent the largest population of mononuclear phagocytes in the intestine and play an important role in the mucosal immune system (4). Inappropriate macrophage activation has been implicated as one of major reasons for failure to resolve acute inflammation in the gut (5). Thus, attention has been focused on controlling dysregulated functions of macrophages, which could augment development of IBD. The relapsing inflammatory disorders are associated with sustained overproduction of pro-inflammatory cytokines in the intestinal lamina propria (6). Intriguingly, in the inflamed gut of patients with IBD, activated macrophages produce significantly more pro-inflammatory cytokines such as TNF-α, IL-23 and IL-6 (7).

The transcription factor STAT3 that plays a key role in inflammation and immunity is activated by IL-6 family cytokines (8). Prolonged or excessive inflammatory response through STAT3 overactivation contributes to chronic inflammation, resulting in an inflammatory disorder and colorectal cancer. Phosphorylation of STAT3 on tyrosine 705 facilitates its dimerization and translocation into nucleus, where it regulates transcription of genes involved in inflammation (9). It has been reported that increased STAT3 phosphorylation is found in the dextran sulfate sodium (DSS)-induced murine colitis model as well as in inflamed colonic mucosa of IBD patients (10, 11). Genome-wide association studies have revealed that STAT3 gene is one of the susceptibility loci in IBD (12). Moreover, persistent activation of STAT3 is implicated in IBD and colorectal cancer (13, 14).

The process of resolution is actively controlled by endogenous anti-inflammatory and pro-resolving mediators (15). Prostaglandins (PGs) are key mediators of inflammation. Their production changes during the different stages of inflammation. PGE_2_ and PGI_2_ are generated to initiate inflammation, while cyclopentenone PGs are produced to terminate inflammation in the later phase of acute inflammation. Cyclopentenone PGs have immunomodulatory and anti-inflammatory effects to switch off inflammatory response. Thus, a shift from PGE_2_ to cyclopentenone PGs contributes to resolution of inflammation (16).

15-Deoxy-△^12,14^-prostaglandin J_2_ (15d-PGJ_2_), a representative J-series cyclopentenone PG, plays important roles in regulating inflammation through inhibition of pro-inflammatory signaling. The biosynthesis of 15d-PGJ_2_ is initiated by cyclooxygenase-2 (COX-2) and subsequently regulated by theprostaglandin D synthase (PGDS) activity. PGDS has two distinct isoforms, hematopoietic PGDS (HPGDS) and lipocalin PGDS (LPGDS). 15d-PGJ_2_ inhibits nuclear factor-kappa B (NF-κB)-mediated expression of vascular cell adhesion molecule 1 and intercellular adhesion molecule 1 in endothelium and the pro-inflammatory enzymes, inducible nitric oxide synthase (iNOS), and COX-2 (17–19). 15d-PGJ_2_ has both anti-inflammatory and pro-resolving activities (20, 21). Although 15d-PGJ_2_ has been reported to protect against acute inflammatory tissue injury (22), detailed molecular mechanisms underlying its pro-resolving effect in intestinal inflammation remain largely unknown. Here, we report that 15d-PGJ_2_ promotes resolution of DSS-induced colitis.

## Materials and Methods

### Animals

Male C57BL/6 mice (5 weeks of age) were purchased from Central Lab. Animal, Inc. (Seoul, South Korea). They were acclimated for 7 days with tap water and a pelleted basal diet before the start of the experiments. The animals were housed in plastic cages under controlled conditions of temperature (23°C ± 2°C), humidity (50% ± 10%), and light (12/12-h light/dark cycle).

### DSS-Induced Colitis

To induce colitis, mice were given drinking water containing 2.5% DSS (MW 36,000–50,000; MP Biomedicals) for 7 days. For evaluation of colitis resolution, mice received normal drinking water for additional 15 days. 15d-PGJ_2_ (2 mg/kg/day) suspended in 10% DMSO in phosphate-buffered saline (PBS) or vehicle was administered intraperitoneally. Mice were euthanized by CO_2_ asphyxiation, and their colorectal parts were taken out, cut longitudinally, and washed with PBS. For histopathological examination, the distal section of colon tissues was fixed in 10% buffered formalin, whereas another portion was flash-frozen in liquid nitrogen and kept at -70°C for further analysis.

### Tissue Lysis and Protein Extraction

Colon tissues were homogenized in an ice-cold lysis buffer [150 mM NaCl, 0.5% Triton-X 100, 50 mM Tris–HCl (pH 7.4), 20 mM EGTA, 1 mM dithiothreitol (DTT), 1 mM Na_3_VO_4_, 1 mM phenylmethylsulfonyl fluoride (PMSF), and EDTA-free protease inhibitor cocktail tablet], followed by a periodical vortex mixing for 30 min at 4°C. The lysates were centrifuged at 14,000 rpm for 15 min at 4°C. The supernatants were collected and stored at - 70°C until use.

### Western Blot Analysis

For Western blot analysis, the total protein concentration was quantified by using the bicinchoninic acid (BCA) protein assay kit (Pierce). Cell lysates (20–50 μg protein) were mixed and boiled in a sodium dodecyl sulfate (SDS) sample buffer for 5 min at 95°C before 8%–17% SDS–polyacrylamide gel electrophoresis (SDS-PAGE). They were separated by SDS-PAGE and transferred to a polyvinylidene difluoride membrane (Gelman Laboratory). The blots were blocked in 5% fat-free dry milk in Tris-buffered saline containing 0.1% Tween 20 (TBST) for 1 h at room temperature. The membranes were incubated for 12 to 24 h at 4°C with primary antibodies for HPGDS, LPGDS, COX-2 (Cayman Chemical), P-STAT3, STAT3 (Cell Signaling Technology), and actin (Sigma Aldrich). Antibodies directed for HPGDS, LPGDS, COX-2, and P-STAT3 were diluted at 1:1,000 and anti-actin antibody was diluted at 1:3,000. The membranes were washed, followed by incubation with 1:3,000 dilution of respective horseradish peroxidase (HRP)-conjugated secondary antibodies (rabbit or mouse) (Zymed Laboratories) for 1 h, and again washed with TBST. Protein expression was visualized with an enhanced chemiluminescence detection kit (Amersham Pharmacia Biotech) and ImageQuant LAS-4000 (GE Healthcare) according to the manufacturer’s instructions.

### Immunohistochemical Analysis and Immunofluorescence Staining

The dissected colon tissues were prepared for immunohistochemical and immunofluorescence analyses of the expression of P-STAT3 and COX-2, respectively. Four to 10 μm sections of 10% formalin-fixed, paraffin-embedded tissues were cut and mounted onto glass slides, deparaffinized three times with xylene and rehydrated through graded alcohol bath. The deparaffinized sections were heated by using microwave and boiled twice for 6 min in 10 mM citrate buffer (pH 6.0) for antigen retrieval. To diminish nonspecific staining, each section was treated with 3% hydrogen peroxide and 4% peptone casein blocking solution for 15 min. For detection of P-STAT3, slides were incubated with anti-P-STAT3 antibody at room temperature for 40 min in Tris-buffered saline containing 0.05% Tween 20, and then developed using respective HRP-conjugated secondary antibodies (rabbit) EnVision™ System (Dako). The peroxidase binding sites were detected by st a ining with 3 ,3′-diaminobenzidine tetrahydrochloride. Finally, counterstaining was performed using Mayer’s hematoxylin. For immunofluorescence analysis of COX-2, the slides were stained with anti-COX-2 antibody in 5% bovine serum albumin at 4°C overnight and then washed 3 times. The slides were then incubated with fluorophore-conjugated secondary antibody (Alexa Fluor 488) for another 1 h at room temperature. Nuclei were counterstained with 4′,6-diamidino-2-phenylindole (DAPI). The fluorescent images were visualized under a confocal microscope.

### Isolation of Murine Lamina Propria Immune Cells From Colonic Tissues

Isolation of colonic lamina propria cells was performed according to the protocol (23). Entire colons from each group were longitudinally cut and washed to remove feces. They were then cut into 1 cm pieces, followed by incubation with predigestion solution containing 5 mM EDTA and 0.145 mg/ml DTT for 20 min at 37°C on a shaking platform. After removal of EDTA by three washes in PBS and passing through a cell strainer (100 µm), the suspension of epithelial, subepithelial, and villus cells were removed. The remaining colon pieces including lamina propria cells and muscle layer were cut by using scissors, and then incubated in digestion media containing 0.05 g of collagenase D (Roche), 0.05 g of DNase I (Sigma-Aldrich) and 0.3 g of dispase II (Roche) for 25 min at 37°C on a shaking platform. After digestion, the lamina propria cells were enriched using Percoll density gradient centrifugation. The resulting cells were then used for flow cytometry analysis.

### Flow Cytometry

Lamina propria cells were stained with a cocktail of antibodies to various markers including CD45, F4/80, CD11b, CD86, CD206, and Gr-1 for 30 mins at 4°C. For intracellular staining, cells were stained with cell surface markers, and incubated Fixation/Permeabilization working solution to each sample for fixation and permeabilization according to the manufacturer’s instructions. The cells were stained with antibody specific to IL-6 for 1 h at 4°C and subjected to flow cytometry. The results were analyzed using FlowJo software

### Preparation of Bone Marrow Derived Macrophages (BMDMs)

Bone marrow cells were isolated from femurs and tibias of C57BL/6 mice. BMDMs are differentiate from bone marrow cells in RPMI1640 medium supplemented with 10% fetal bovine serum and 20 ng/ml M-CSF for 7 days. After removal of non-adherent cells, BMDMs were detached from the plate using Accutase (Innovative Cell Technologies) and used for the further experiments. To induce M1 macrophage polarization, BMDMs were stimulated with bacterial lipopolysaccharide (LPS; 100 ng/ml) for 4 h.

### Real-Time RT-PCR

Total RNA was isolated from BMDM or mouse colon tissues using TRizol^®^ according to the manufacturer’s instruction. RNA was then used to synthesize complementary DNA (cDNA) and further analyzed by using RealHelix qPCR kit (Nanohelix) with Applied Biosystems 7500 Fast Real-Time PCR System following the manufacturer’s instructions.

### Statistical Analysis

All data are expressed as means ± SD of at least three independent experiments, and statistical analysis was performed using ANOVA. The criterion for statistical significance was *p < 0.05, **p < 0.01, and ***p < 0.001.

## Results

### 15d-PGJ_2_ Is Produced During Resolution of Intestinal Inflammation

**Figure 1:**
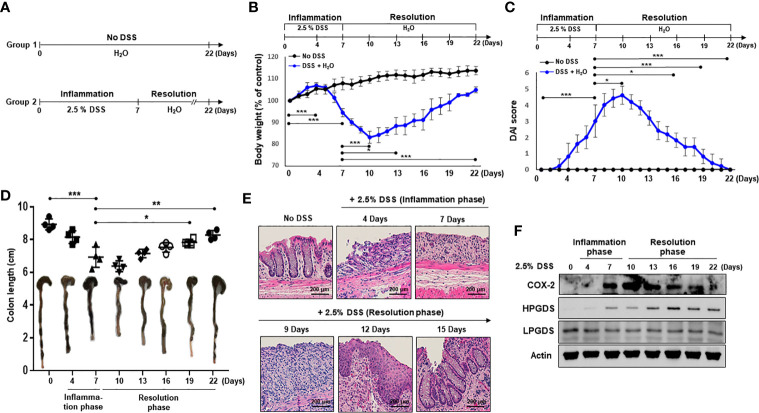
HPGDS responsible for the synthesis of 15d-PGJ_2_ is transiently upregulated during spontaneous resolution of dextran sulfate sodium (DSS)-induced colitis. **(A)** Mice were administrated with drinking water containing 2.5% DSS for 7 days, followed by normal water for 15 days. “No DSS” indicates mice without DSS administration. **(B)** Gradual change of body weight. **(C)** Disease activity index (DAI) based on the severity of stool consistency and rectal bleeding was measured every day during the experiment period. **(D)** The comparison of the colon length at the inflammation phase and resolution phase of intestinal inflammation. **(E)** Representative distal colon sections stained with hematoxylin and eosin (H&E). **(F)** COX-2, HPGDS, and LPGDS levels in colon tissue of the inflammation phase and resolution phase of inflammation were determined by immunocytochemical analysis. All data represent mean ± S.D. (n = 4). ^*^ *p* < 0.05, **p < 0.01, and ^***^ *p* < 0.001.

To investigate resolution of intestinal inflammation, the DSS-induced colitis model was used. Mice were given drinking water containing 2.5% DSS for 7 days, which causes severe inflammatory damage to colonic epithelium (24). Mice were then exposed to normal drinking water for an additional 15 days to allow intestinal inflammation to subside (**Figure 1A**). Mice began to lose body weight after 5 days of DSS treatment, and the body weight loss continued 3 days following termination of DSS exposure (**Figure 1B**). Mice restored the body weight from day 11. The weight loss of the mice associated with intestinal inflammation was completely recovered after 15 days of drinking normal water (**Figure 1B**). DSS-treated mice demonstrated acute colitis with massive colon ulceration, crypt damage, and severe inflammation. Based on the severity of stool consistency and rectal bleeding, pathogenic conditions were scored from 0 to 3. The sum was given into a form of the disease activity index (DAI). Mice administered DSS exhibited severe symptoms with liquid stool and large amount of rectal bleeding. DAI score reached a maximal level on day 10, and gradually decreased during the resolution phase (**Figure 1C**). In addition, mice exposed to DSS displayed shortening of the colon length, but this was mostly recovered by the end of experiment (**Figure 1D**). We also performed histological evaluation of dysplasia in the colonic mucosa by hematoxylin and eosin (H&E) staining. Exposure to DSS almost completely destroyed the architecture of colonic mucosa, but this was eventually recovered (**Figure 1E**).

15d-PGJ_2_ is formed from arachidonic acid during inflammatory response (25). To assess the possible involvement of 15d-PGJ_2_ in adaptive survival response to DSS-induced colitis, the expression of HPGDS and LPGDS as well as COX-2 was measured during onset and resolution of inflammation. COX-2 expression was increased in the late phase of inflammation, and decreased thereafter during the resolution phase of inflammation (**Figure 1F**). Notably, HPGDS expression was significantly upregulated, while LPGDS expression was barely changed during the resolution of inflammation (**Figure 1F**).

### Inhibition of HPGDS Exacerbates DSS-Induced Colitis

**Figure 2:**
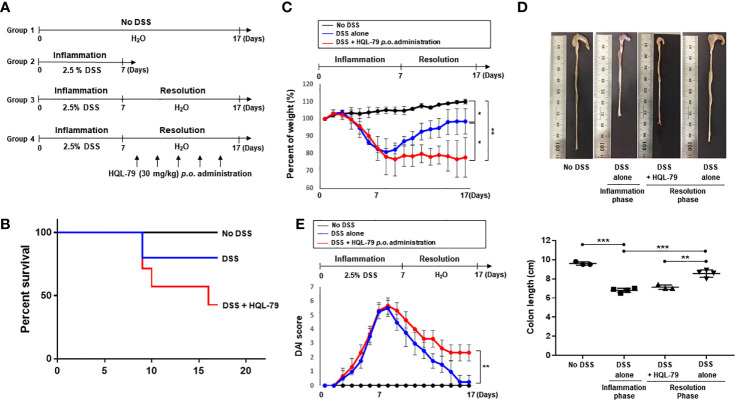
Inhibition of HPGDS leads to the failure in resolution of DSS-induced intestinal inflammation. **(A)** Mice were given with drinking water containing 2.5% DSS for 7 days, followed by normal water for another 10 days. HQL-79 (30 mg/kg) was administrated orally for 10 days every other day. **(B)** The survival rate, **(C)** the change in the body weight, and **(D)** DAI score were monitored every day during the experiment period. The survival rate of the mice was expressed by using Kaplan–Meier plot. **(E)** The colon length was measured when mice were sacrificed. All data represent mean ± S.D. (n = 3 or 4). ^*^ *p* < 0.05, ^**^ *p* < 0.01, and ^***^ *p* < 0.001.

HPGDS converts PGH_2_ formed by COX-2 to PGD_2_, an intermediate in 15d-PGJ_2_ biosynthesis. The increased expression of HPGDS hence results in enhanced production of 15d-PGJ_2_, which plays an essential role in regulating immune response for its cytoprotective effects (26, 27). In order to determine whether the pro-resolving effects of endogenous 15d-PGJ_2_ on DSS-induced colitis attributable to HPGDS, we utilized HQL-79, a commonly used inhibitor of this enzyme (28, 29). The mice were given 2.5% DSS in drinking water *ad libitum* for 10 days. HQL-79 (30 mg/kg) was given *via* gavage during the subsequent resolution phase (**Figure 2A**). Under these conditions, HQL-79 treated mice exhibited a significant decrease in survival (**Figure 2B**). In addition, pharmacologic inhibition of HPGDS activity severely compromised resolution of intestinal inflammation. Thus, HQL-79 administration abrogated recovery of body weight (**Figure 2C**) and aggravated the severity of diarrhea and rectal bleeding (**Figure 2D**). Furthermore, mice treated with the HPGDS inhibitor were not able to recover DSS-induced shortening of the colorectal length, compared with those treated with vehicle (**Figure 2E**). These data demonstrate that 15d-PGJ_2_ plays an important role in resolution of intestinal inflammation.

### 15d-PGJ_2_ Promotes Recovery of DSS-Induced Colitis in Mice

**Figure 3:**
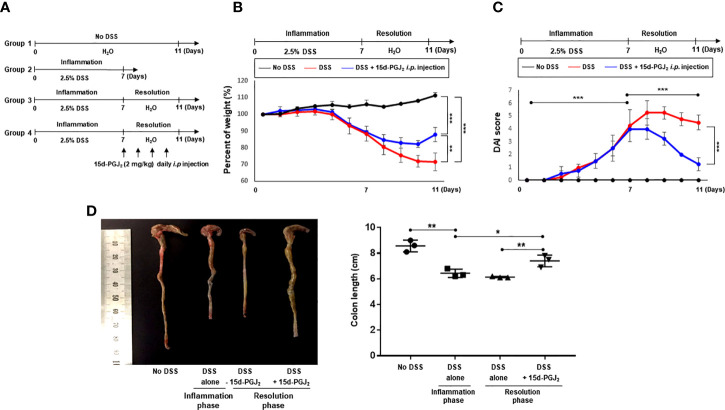
15d-PGJ_2_ ameliorated pathological symptoms for resolution of intestinal inflammation. **(A)** Mice were administrated with drinking water containing 2.5% DSS for 7 days, followed by normal drinking water for 4 days. 15d-PGJ_2_ (2 mg/kg) or vehicle was intraperitoneally injected to mice every day during resolution phase of inflammation. **(B)** The change of body weight was monitored and **(C)** DAI was scored. **(D)** On day 7 (inflammation phase) and day 11 (resolution phase), the colon length was determined, compared with no DSS group. All data represent mean ± S.D. (n=3). ^*^ *p* < 0.05, ^**^ *p* < 0.01, and ^***^ *p* < 0.001.

Next, we assessed the effect of exogenous 15d-PGJ_2_ on the resolution of DSS-induced colitis. For this purpose, mice were treated with 2.5% DSS in drinking water *ad libitum* for 7 days and then divided into two groups. One group of mice was allowed to resolve intestinal inflammation for an additional 4 days on normal drinking water, while the other group received 15d-PGJ_2_ (2 mg/kg) 4 times intraperitoneally during the same period (**Figure 3A**). Mice administered 15d-PGJ_2_ exhibited significantly improved body weight recovery, compared with mice given vehicle (**Figure 3B**). In addition, 15d-PGJ_2_ administration ameliorated the severity of diarrhea and rectal bleeding (**Figure 3C**) and recovered DSS-induced shortening of the colorectal length (**Figure 3D**). These data suggest that 15d-PGJ_2_ stimulates resolution of the DSS-induced colitis in mice.

### 15d-PGJ_2_ Induces Macrophage Polarization During Resolution of DSS-Induced Murine Colitis

Macrophages play a key role in controlling the onset and the resolution of acute inflammation (30). Macrophages are classified into two types, classically activated (M1) macrophages and alternatively activated (M2) macrophages. The M1 macrophages produce pro-inflammatory cytokines and reactive nitrogen and oxygen intermediates, resulting in inflammation. In contrast, the M2 macrophages have anti-inflammatory and pro-resolving properties, which are involved in enhancement of phagocytic activity, tissue remodeling, etc (31). As an initial step towards investigating the effects of 15d-PGJ_2_ on mobilization of macrophages, leukocytes infiltrated to the lamina propria (CD45^+^) were isolated from colon tissue as depicted in **Supplementary Figure 1A**. While the proportion of infiltrated leukocytes (CD45^+^) was significantly increased as a consequence of intestinal inflammation caused by DSS, this was decreased during the resolution phase following 15d-PGJ_2_ treatment (**Supplementary Figure 1B**).

**Figure 4:**
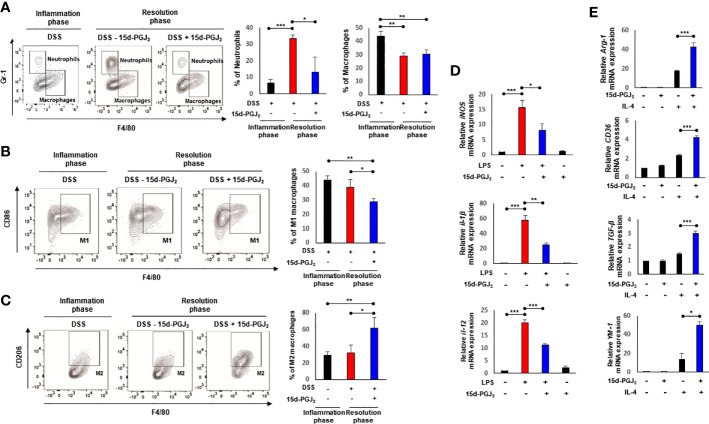
15d-PGJ_2_ induces leukocyte infiltration and macrophage polarization. Lamina propria immune cells were isolated from the colons of DSS-treated mice, collected on day 7 (Inflammation phase) and day 11 (Resolution phase) as described in *Materials and Methods*. **(A–C)** The percentage of neutrophils (CD45^+^ CD11b^+^ Gr-1^+^ F4/80^-^) macrophages (CD45^+^ CD11b^+^ Gr-1^-^ F4/80^+^), M1 macrophages (CD45^+^ CD11b^+^ Gr-1^-^ F4/80^+^ CD86^+^), and M2 macrophages (CD45^+^ CD11b^+^ Gr-1^-^ F4/80^+^ CD206^+^) was determined by flow cytometery. The graphs show the percentage of each cell population. **(D)** BMDMs were treated with LPS (100 ng/ml) in the absence or presence of 15d-PGJ_2_ (10 μM). The mRNA levels of M1 markers including *iNOS, IL-1β*, and *IL-12* were measured by real-time PCR. **(E)** BMDMs were co-treated with IL-4 and 15d-PGJ_2_ for 4 h. The mRNA levels of M2 markers including *Arg-1*, *CD36*, *TGF-β*, and *YM-1* were determined by real-time PCR. All data represent mean ± S.D. (n=3). ^*^ *p* < 0.05, ^**^ *p* < 0.01, and ^***^ *p* < 0.001.

Next, we determined the proportion of macrophages by flow cytometry. The number of neutrophils (CD45^+^ CD11b^+^ Gr-1^+^ F4/80^-^) in lamina propria of 15d-PGJ_2_-treated mice decreased significantly, compared with the vehicle control. The proportion of macrophages (CD45^+^ CD11b^+^ Gr-1^-^ F4/80^+^) was elevated during the inflammatory phase, but reduced in both groups during the resolution phase (**Figure 4A**). Considering the differential roles for macrophages in the inflammation and its resolution, we then measured the proportions of M1 and M2 macrophages of colonic mucosa by flow cytometry. As shown in **Figure 4B**, 15d-PGJ_2_ administration lowered the proportion of M1 macrophages (CD45^+^ CD11b^+^ Gr-1^-^ F4/80^+^ CD86^+^) while it significantly enhanced that of M2 macrophages (CD45^+^ CD11b^+^ Gr-1^-^ F4/80^+^ CD206^+^) during the resolution of intestinal inflammation (**Figure 4C**).

Macrophages that infiltrate the inflamed tissue are derived from bone marrow precursors that migrate *via* the peripheral circulation. To further examine the effect of 15d-PGJ_2_ on macrophage polarization more systematically, BMDMs isolated from the same strain of mice used for inducing colitis were stimulated with LPS, which has been known to induce the M1 polarization. LPS treatment upregulated the mRNA expression of M1 markers including *iNOS*, *IL-1β*, and *IL-12*. 15d-PGJ_2_ (10 μM) significantly inhibited the expression of M1 markers in LPS-stimulated macrophages (**Figure 4D**). In addition, we investigated whether 15d-PGJ_2_ could affect M2 macrophage polarization. 15d-PGJ_2_ alone could not significantly induce expression of M2 macrophage markers, but synergistically increased M2 macrophage polarization when BMDMs were co-treated with IL-4, a known inducer of M2 makers (**Figure 4E**). These findings suggest that 15d-PGJ_2_ regulates leukocyte trafficking and renders macrophages polarized into the M2 phenotype while it suppresses manifestation of the M1 phenotype.

### 15d-PGJ_2_ Inhibits IL-6 Expression and STAT3 Activation During Resolution of DSS-Induced Intestinal Inflammation

**Figure 5:**
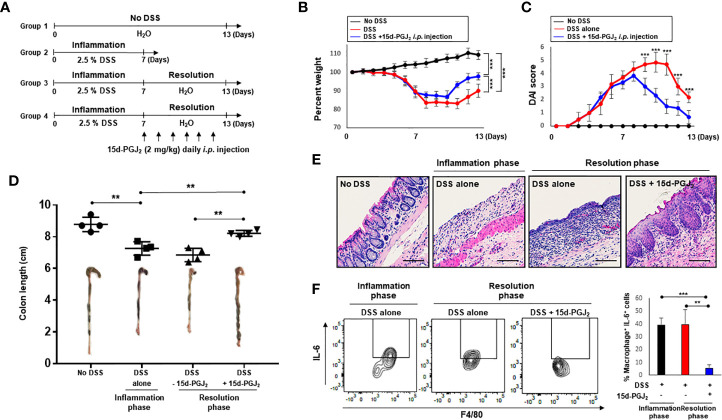
15d-PGJ_2_ treatment administration accelerates the resolution of colitis and inhibits IL-6 expression in intestinal macrophages of DSS-treated mice. **(A)** Mice were administrated with drinking water containing 2.5% DSS for 7 days, followed by normal water for 6 days during which 15d-PGJ_2_ (2 mg/kg) or vehicle was intraperitoneally injected on daily basis. Gradual change of body weight **(B)** and DAI. **(C)** were measured every day during the experiment period. **(D)** The comparison of the colon length at the inflammation phase and resolution phase of intestinal inflammation. **(E)** Representative distal colon sections stained with H&E. Scale bar, 200 μm. **(F)** Intestinal macrophages were collected on day 7 or day 13 from DSS-treated mice, and the proportion of macrophages expressing IL-6 (CD45^+^ CD11b^+^ Gr-1^−^ F4/80^+^ IL-6^+^) was measured by flow cytometry. *, **, *** Significantly different between the groups compared (*p < 0.05, **p < 0.01, and ***p < 0.001).

Increased pro-inflammatory cytokine production is a hallmark of intestinal inflammation (32, 33). In particular, overproduction of IL-6 has been reported to trigger chronic intestinal inflammation and subsequently colon cancer. In order to determine whether the pro-resolving effects of 15d-PGJ_2_ are associated with inhibition of IL-6 in macrophages, mice were given 2.5% DSS in drinking water for 7 days, followed by normal water for another 6 days to allow resolution of acute intestinal inflammation (**Figure 5A**). When the mice were given normal water, 15d-PGJ_2_ was administrated on daily basis into peritoneum of mice. DSS-induced intestinal inflammation were significantly ameliorated in 15d-PGJ_2_-treated mice, as indicated by the improvement of body weight loss (**Figure 5B**), DAI score (**Figure 5C**), shortening of colon length (**Figure 5D**), and disruption of crypt architecture (**Figure 5E**). Next, macrophages expressing IL-6 were selectively identified by flow cytometry. 15d-PGJ_2_ inhibited the proportion of macrophages expressing IL-6 (CD45^+^ CD11b^+^ Gr^−1^ F4/80^+^ IL-6^+^) during resolution of intestinal inflammation (**Figure 5F**).

**Figure 6:**
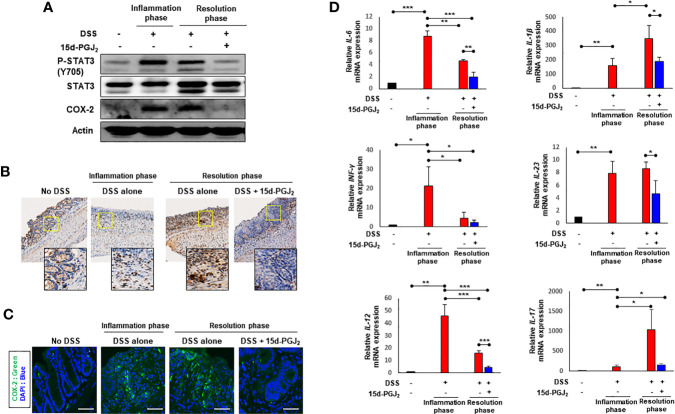
15d-PGJ_2_ inhibited DSS-induced STAT3 phosphorylation and expression of COX-2 and pro-inflammatory cytokines in mouse colon. Colon tissue were collected on day 7 (inflammation phase) and day 13 (resolution phase). **(A)** The levels of P-STAT3 (Y705) and COX-2 were determined by immunoblot analysis. Actin was used as an equal loading control for normalization. **(B)** Immunohistochemical detection of P-STAT3 (brown spots) in colon tissue were determined. To verify the expression of COX-2 in colon tissue, **(C)** immunofluorescence analysis was conducted using anti-COX-2 antibody. Scale bar, 200 μm. **(D)** Pro-inflammatory cytokines including *IL-6, IL-1β, INF-γ, IL-23, IL-12*, and *IL-17* were determined in colon tissue by real-time PCR. *, **, *** Significantly different between the groups compared (*p < 0.05, **p < 0.01, and ***p < 0.001).

STAT3, a representative transcription factor involved in intestinal inflammation, is mainly activated by IL-6 (34). One of the key events in activation of STAT3 is its phosphorylation on the tyrosine 705 (Y705) residue. 15d-PGJ_2_ administration given during the resolution phase markedly inhibited DSS-induced phosphorylation of STAT3 in colonic mucosa (**Figure 6A**). It also abolished the DSS-induced expression of COX-2 (**Figure 6A**), a principal pro-inflammatory enzyme frequently overexpressed in the inflamed colonic mucosa of patients with IBD (35, 36). The inhibitory effects of 15d-PGJ_2_ on DSS-induced phosphorylation of STAT3 (**Figure 6B**) and COX-2 expression (**Figure 6C**) were verified by immunohistochemical analysis and immunofluorescence staining, respectively. Likewise, expression of representative pro-inflammatiry cytokines was significantly reduced by 15d-PGJ2 administered during the resolution phase (**Figure 6D**).

## Discussion

Acute inflammation is a protective immune reaction against microbial infection and tissue injury (37). After its completion of critical function in host defense, acute inflammation should be properly resolved to avoid chronic inflammation responsible for the pathogenesis of many prevalent disorders, such as cancer, obesity, and IBD (38). In this study, we investigated the resolution of intestinal inflammation by using a DSS-induced murine colitis model that mimics the characteristics of human IBD (2, 39). The onset and termination of acute intestinal inflammation are coordinately regulated by endogenous checkpoints to avoid progression to chronic inflammation. The two contrasting processes are finely orchestrated by endogenous pro-inflammatory and pro-resolving mediators (40, 41).

PGs are key lipid mediators/modulators derived from arachidonic acid that have diverse functions in resolution of inflammation. It has been reported that some of pro-inflammatory PGs generated in inflamed tissue amplify acute inflammation, whereas anti-inflammatory/pro-resolving PGs, such as 15d-PGJ_2_, promote proper termination of inflammation (26, 42–44). Although 15d-PGJ_2_ is detected in self-resolving exudates, the molecular mechanism underlying its pro-resolving effects on intestinal inflammation remains poorly understood. In our present study, pharmacologic inhibition of 15d-PGJ_2_ production impaired resolution of DSS-induced murine colitis. Conversely, administration of exogenous 15d-PGJ_2_ promoted resolution of intestinal inflammation through its immunomodulatory and anti-inflammatory effects.

Resolution of inflammation is an active process that requires inhibition of further leukocyte recruitment and elimination of leukocytes from inflamed sites. As part of the gut inflammatory response, neutrophils recruited to the inflamed site are activated and undergo oxidative burst, a critical event in the host defense. This leads to overproduction of reactive oxygen species (ROS) with which the neutrophils kill and eliminate the infectious pathogens (45–47). Therefore, infiltrated neutrophils are essential for host defense against invading pathogens, but excessive neutrophil infiltration is limited and neutrophils eventually die *via* apoptosis. Accelerated neutrophil apoptosis has severe pathological consequences such as infection and autoimmune diseases (48–50). On the other hand, delayed neutrophil apoptosis causes inflammatory disorders like chronic pulmonary obstructive disease, and rheumatoid arthritis (51–53).

It has been reported that 15d-PGJ_2_ promotes endothelia cell apoptosis as well as granulocyte apoptosis (54, 55). Furthermore, 15d-PGJ_2_ induces synoviocyte apoptosis and suppresses arthritis in rats (55). In this study, we found that 15d-PGJ_2_ inhibited neutrophil infiltration during the resolution phase of inflammation. We speculate that 15d-PGJ_2_ might regulate not only excessive neutrophil infiltration into inflamed area, but also neutrophil apoptosis, thereby promoting the resolution of intestinal inflammation.

IBD is characterized by persistent infiltration of inflammatory immune cells including neutrophils and macrophages within the gut and in the circulation (56). In particular, intestinal macrophages contribute to the gut homeostasis by balancing pro-inflammatory and anti-inflammatory cytokines (57). The M1 macrophages produce pro-inflammatory cytokines and oxidative stress, resulting in initiation and progression of inflammation, while the M2 macrophages exert anti-inflammatory and pro-resolving functions, thereby promoting resolution of inflammation (31). It has been reported that phenotypic switching from M1 macrophages to the M2 macrophages plays a critical role in the resolution of inflammation and tissue repair (58, 59). The number of M1 macrophages which release pro-inflammatory cytokines is increased in the colonic tissue of IBD patients (59–61). We have found that 15d-PGJ_2_ counteracts excessive inflammatory responses and stimulates resolution of colitis in the colon of DSS-treated mice by regulating macrophage polarization. However, macrophage polarization during different phases of the inflammatory and resolving processes is not a fixed but a dynamic process in which macrophages exhibit a high degree of plasticity, existing in different intermediate forms of activation (62). Therefore, more precise assessment of the profiles of different forms of macrophages merits further investigation to better understand the 15d-PGJ_2_-mediated resolution of intestinal inflammation.

A diverse of transcription factors including STAT3, NF-κB, AP-1, and PPARγ are involved in M1 or M2 macrophage polarization (63). PPARγ plays a key role in inflammation by regulating both M1 and M2 polarization. PPARγ negatively regulated NF-κB and AP-1 signaling, resulting in inhibition of M1 macrophage polarization. In contrast, PPAR γ was also found to mediate M2 polarization by inducing *Arg-1* and *CD36* expression (63, 64). Though the role for PPARγ in IBD is controversial (65), a plethora of studies suggest that PPARγ activation might impede pathophysiological imbalances associated with IBD (66).15d-PGJ_2_, as an endogenous PPARγ ligand, has been reported to inhibit pro-inflammatory signaling (16, 67). Pretreatment with PPARγ agonists 15d-PGJ_2_ and rosiglitazone prevented acute stress-induced colonic inflammation and barrier dysfunction in rats, and these effects were reverted by a PPARγ specific antagonist (68). However, a recent study by Takagi and colleagues reveals that 15d-PGJ_2_ inhibits development of intestinal inflammation in mice *via* PPARγ-independent and Nrf2-heme oxygenase-dependent mechanisms (69).

Overproduction of IL-6 has been considered as a result of failure in resolution of intestinal inflammation. It is clearly defined that incomplete intestinal resolution leads to IBD (5, 70). IL-6 production by lamina propria macrophages and CD4^+^ T cells was increased in experimental colitis and in patients with IBD (71, 72). Blockade of IL-6 signaling with a neutralizing monoclonal antibody was effective in suppressing chronic intestinal inflammation in mouse models (71, 73). In the present study, we notably found that intraperitoneal injection of exogenous 15d-PGJ_2_ reduced the proportion of macrophages expressing IL-6. 15d-PGJ_2_ contains a reactive α,β-unsaturated carbonyl present in the cyclopentenone ring and hence can form covalent adducts with the cysteine thiol of intracellular regulatory proteins (74, 75). We have recently reported that 15d-PGJ_2_ covalently binds to the cysteine 259 of STAT3 and inhibits its activation (76). We also found that COX-2 expression during the resolution period was inhibited by administration of exogenous 15d-PGJ_2_. This may be ‘resolution-toxic’ (40) as the COX-2 activity is responsible for the production of PGD_2_, a precursor of 15d-PGJ_2_. Therefore, it is likely that excessive amounts of 15d-PGJ_2_ can block the further synthesis of this proresolving mediator, constituting a negative-feedback loop for the self-limited resolution.

**Figure 7:**
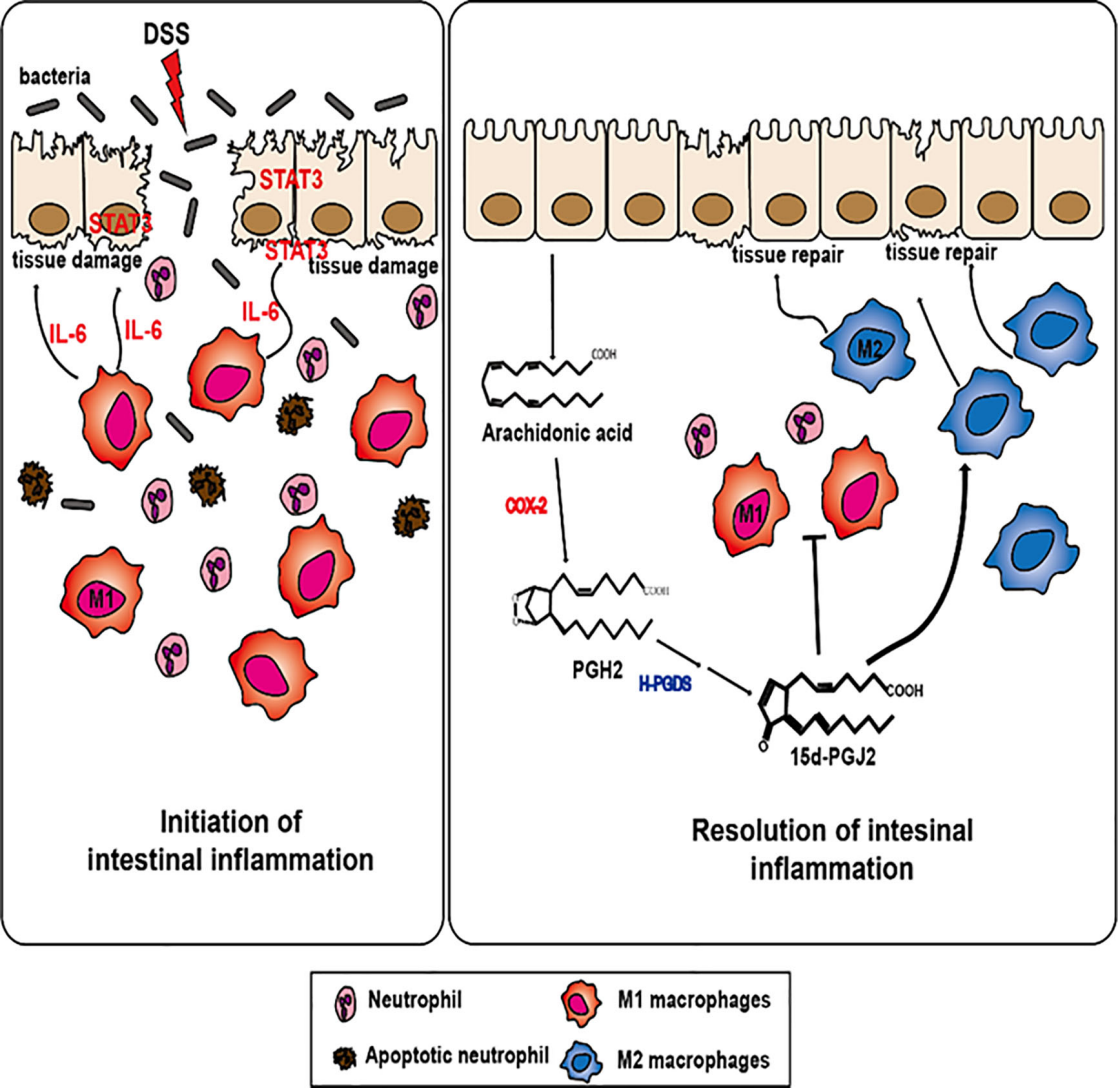
A proposed mechanism underlying the pro-resolving effects of 15d-PGJ_2_ on resolution of intestinal inflammation. 15d-PGJ_2_, is endogenously generated from arachidonic acid induced by COX-2 and subsequent HPGDS during resolution of intestinal inflammation. 15d-PGJ_2_ promotes intestinal inflammation through regulating macrophage polarization.

In conclusion, our findings suggest that 15d-PGJ_2_, generated during inflammatory process, stimulates the resolution of experimentally induced intestinal inflammation by inducing M2 macrophage polarization (**Figure 7**). 15d-PGJ_2_ administration decreased the proportion of M1 macrophages and macrophages expressing IL-6, which accounts for its inhibition of STAT3 activation. Unresolved inflammation caused by inappropriate macrophage polarization can result in inflammation-associated disorders like IBD, diabetes, and arthritis (63, 77). Regulating macrophage polarization is a crucial process in resolving inflammation, thereby, preventing the development of chronic inflammatory disorders. 15d-PGJ_2_, generated during resolution of intestinal inflammation, is one of the prime endogenous proresolving molecules and this lipid mediator might have a therapeutic potential in the management of IBD associated with macrophage dysfunction.

## Data Availability Statement

The original contributions presented in the study are included in the article/**Supplementary Material**; further inquiries can be directed to the corresponding author.

## Ethics Statement

The animal study was reviewed and approved by Institutional Animal Care and Use Committee at Seoul National University, South Korea.

## Author Contributions

WK conceived and designed the study. J-HJ, XZ, and HS provided the technical support. WK drafted the article. Y-JS supervised the work and edited the manuscript. All authors contributed to the article and approved the submitted version.

## Funding

This study was supported by the Global Core Research Center (GCRC) grant (No. 2011-0030001) and the BK21 FOUR Program (5120200513755) from the National Research Foundation, Republic of Korea.

## Conflict of Interest

The authors declare that the research was conducted in the absence of any commercial or financial relationships that could be construed as a potential conflict of interest.

## Supplementary Material

Supplementary Figure 1 Gating for lamina propria immune cells. Lamina propria immune cells were isolated from the colon of no DSS group, inflammation phase group and resolution phase group. (A) Lamina propria immune cells are stained with CD45, CD11b, Gr-1, F4/80, CD206, CD86 antibodies and then gate on the cells of interest according to the manufacturer’s protocol. (B) The number and the percentage of total immune cells (CD45^+^) in the lamina propria of mice was determined by flow cytometry. All data represent mean ± S.D. (n=3), **p* < 0.05 and ****p* < 0.001.
