# Supplementary figures and images for: 15-Deoxy-△12,14-Prostaglandin J2 Promotes Resolution of Experimentally Induced Colitis
Source: Front Immunol. 2021 Feb 2;12:615803. doi: 10.3389/fimmu.2021.615803 (PMC7901909; doi:10.3389/fimmu.2021.615803)

**A**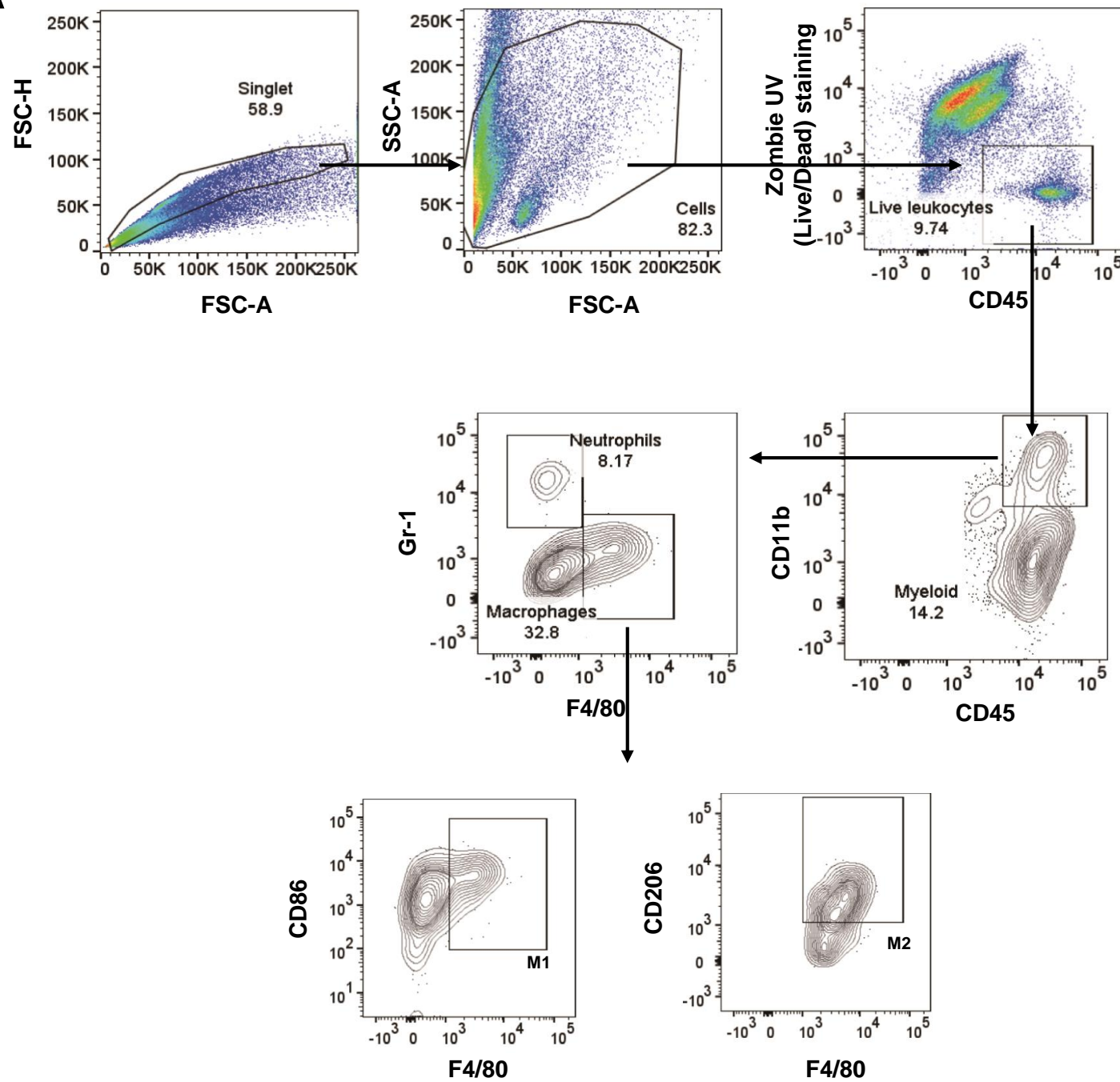

**B**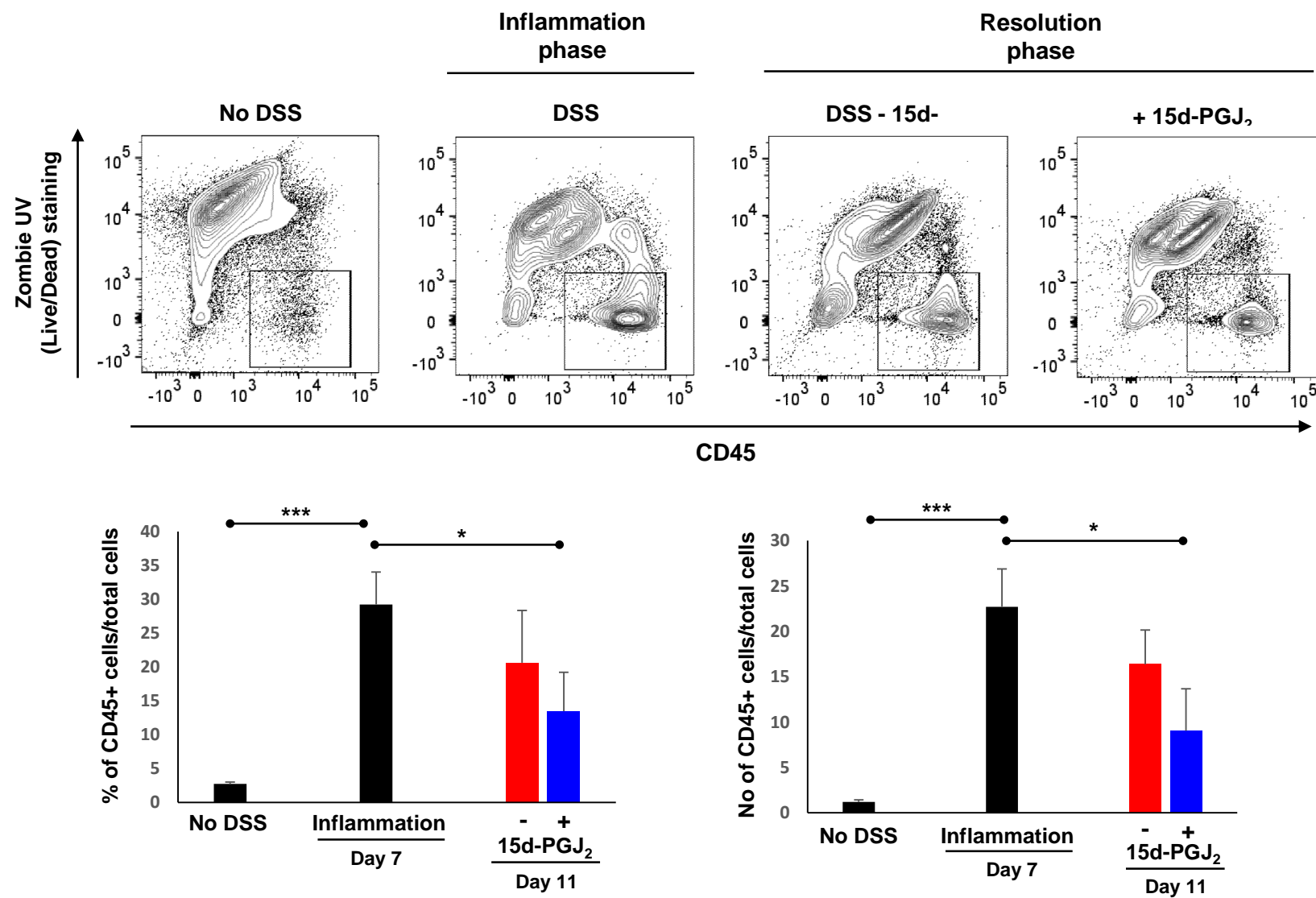

Supplement: Supplementary Figure 1 — Gating for lamina propria immune cells. Lamina propria immune cells were isolated from the colon of no DSS group, inflammation phase group and resolution phase group. (A) Lamina propria immune cells are stained with CD45, CD11b, Gr-1, F4/80, CD206, CD86 antibodies and then gate on the cells of interest according to the manufacturer’s protocol. (B) The number and the percentage of total immune cells (CD45+) in the lamina propria of mice was determined by flow cytometry. All data represent mean ± S.D. (n=3), *p < 0.05 and ***p < 0.001. [file DataSheet_1.pdf]
